# Mechanical chest compressions in the coronary catheterization laboratory – do not hesitate to go step further!

**DOI:** 10.1186/s13049-016-0293-5

**Published:** 2016-08-17

**Authors:** Jan Bělohlávek, Tomáš Kovárník

**Affiliations:** 2nd Department of Medicine, Cardiovascular Medicine, 1st Faculty of Medicine, Charles University in Prague and General University Hospital in Prague, U Nemocnice 2, Prague 2, 128 00 Czech Republic

## Abstract

Authors Wagner et al. in your journal demonstrated effectiveness of mechanical chest compressions in the coronary catheterization laboratory to facilitate coronary intervention and survival in patients requiring prolonged resuscitation efforts. We dare to comment on this article and advocate to use mechanical chest compressions only as a bridge to extracorporeal membrane oxygenation to completely substitute failed circulation and enable percutaneous coronary intervention or other procedures to treat the cause of cardiac arrest.

## Dear editors,

In line with an increasing interest in rescuing patients from refractory cardiac arrest by means of routine implementation of mechanical devices, we have read with a great interest an article by Wagner et al. [1] in your journal. Authors have to be applauded and congratulated for their longterm efforts in instituting and analysing feasibility and effectivity of using mechanical chest compressions in the cathlab [2, 3]. As nicely shown in their recent study, candidates for prolonged cardiopulmonary resuscitation (CPR) entering cathlab at certain point of their illness recruit from different groups of patients: in hospital cardiac arrests, out of hospital cardiac arrests (OHCA) brought directly to cathlab or via emergency department, STEMIs, nonSTEMIs, elective patients, cardiogenic shock patients with failing circulation, occasionally also postoperative cardiac surgery patients or even others. In present series, 62 % of patients entered the cathlab in cardiogenic shock, ie. with failing circulation, but still, with circulation! Accordingly, significant proportion of patients at the time of cardiac arrest with a need of prolonged CPR has been already cannulated with available vascular access for coronary intervention. During cardiac arrest, the main obstacle for effective invasive treatment may be the establishment of vascular access. To cannulate femoral vessels during ongoing chest compressions may be challenging even for highly trained invasive cardiologists and in case of femoral puncture failure, other approach like axillary is at least same difficult or even more demanding. Therefore, in harmony with the authors’ point in discussion and as routinely practiced in our cathlab, we strongly advocate to proceed one step further and use mechanical chest compressions only for a limited period of time before venoarterial ECLS/ECMO (extracorporeal life support/membrane oxygenation) is instituted to provide adequate organ perfusion. At our cathlab, preassembled ECLS device is prepared 24/7 and wet priming takes far below 10 min in hands of a perfusionist, who is also available 24/7. Consequently, majority of our patients entering cathlab under ongoing CPR has been successfully put on ECMO in less than 15 min since cathlab admission. Subsequent coronary or other intervention, ie. pulmonary catheterization embolectomy or further diagnostic angiographies may proceed in much less stressful conditions and with better angiographic accuracy. In case of already established vascular access, mentioned implantation times may be even substantionaly shortened. Definitely, benefits of extracorporeal circulation may in certain cases be outweighted by vascular or other inherent complications. Nonetheless, favorable outcome patients in current study by Wagner et al. had cardiac arrests exactly within the range of 15 min, suitable for establishing the extracorporeal circulation. Whether such an approach, at least in OHCA patients, is feasible, safe and beneficial, might hopefully elucidate our ongoing trial [4]. In other cases, it just seems to be reasonable.
